# Perioperative management in a patient with type 1 diabetes mellitus who presented severe hypoglycemia during dental implant surgery: a case report

**DOI:** 10.1186/s12903-018-0679-z

**Published:** 2018-12-07

**Authors:** Hajime Shimoda, Tetsu Takahashi

**Affiliations:** Division of Oral and Maxillofacial Surgery, Department of Oral Medicine and Surgery, Tohoku University Graduate School of Dentistry, 4-1 Seiryomachi, Aoba-ku, Sendai, Miyagi 980-8575 Japan

**Keywords:** Dental implant surgery, Type 1 diabetes mellitus, Severe hypoglycemia, Perioperative glycemic management

## Abstract

**Background:**

Patients with type 1 diabetes mellitus (DM) have poor glycemic control owing to extreme impairments in glucose tolerance. There are few reports regarding dental implant surgery in patients with type 1 DM. We describe herein the perioperative glycemic management in an outpatient with type 1 DM who experienced a rare case of severe hypoglycemia during dental implant surgery. Only one such case has previously been reported.

**Case presentation:**

A 60-year-old male patient diagnosed with type 1 DM was scheduled for dental implant primary surgery. Premedication with peroral antibiotics was carried out to prevent possible systemic infection as a complication of DM. The patient was treated to control intraoperative hypertension with diligent attention to cardiovascular conditions by using a bolus administration of nicardipine and diltiazem. During surgery, he abruptly complained of hypoglycemic symptoms and had a blood glucose level of 32 mg/dL. Following oral administration and electrolyte-combined infusion of glucose, he immediately recovered from the critical situation. The surgical procedure, involving a lower jaw implant fixture placement, was performed as planned and resulted in less invasion, limited to the area of implant fixture placement within the right mandibular region of the two molars, compared to implant surgery that spans the entire lower jaw.

**Conclusions:**

The present case suggests that it is essential to promptly monitor possible signs of hypoglycemia-precipitated acute symptoms in patients with DM. In addition, it is also necessary to appropriately administer insulin with an electrolyte-combined infusion of glucose for deliberate glycemic control; this is particularly true in patients with type 1 DM undergoing relatively highly-invasive oral surgical manipulation such as commonly performed dental implant surgery spanning the entire jaw.

## Background

The frequency of opportunities regarding perioperative systemic management for medically compromised patients with diabetes mellitus (DM) is steadily increasing in oral and maxillofacial surgical ambulatory care units. In particular, patients with type 1 DM exhibit poor glycemic control owing to an extreme impairment of glucose tolerance based on insufficient insulin secretion. On the other hand, for patients with diabetes who are undergoing surgery, appropriate glycemic control throughout the perioperative period needs to be maintained to conserve the endocrine-metabolic balance between insulin and hyperglycemia-promoting hormones, such as cortisol and adrenaline. In this report, we discuss perioperative management, including glycemic control, in a dental implant outpatient with type 1 DM.

A case with impending hypoglycemia masked by post-extraction labial paresthesia has been reported in a patient with type 1 DM [1]. However, there are few reports regarding dental implant surgery in patients with type 1 DM. Therefore, the present case is rare or unusual case with respect to an occurrence of acute severe hypoglycemia during dental implant surgery. Thus, this report contributes to the literature in this area. Further, we think that this paper will be of interest to the readership of journal because it raises awareness of the importance of preoperative planning for potential hypoglycemic episodes in patients with type 1 DM.

### Case presentation

A 60-year-old male patient (height: 170 cm, weight: 60 kg) diagnosed with type 1 DM was scheduled for dental implant primary surgery in the right mandibular first and second molar region. The present patient, who had diabetic nephropathy and retinopathy as secondary complications, was prescribed intensification therapy of subcutaneous injection of insulin (ultra-rapid-acting insulin aspart/long-acting insulin glargine). The patient’s glycated hemoglobin (HbA1c) level was 6.4%, but he exhibited large and irregular diurnal variations in blood glucose values. Preoperative blood biochemistry examination revealed elevated alkaline phosphatase (492 U/L) and creatine kinase (282 U/L) and decreased albumin (3.6 g/dL) and glucose (39 mg/dL), accompanied by few subjective hypoglycemic symptoms such as nausea, malaise, and drowsiness. Urinary ketone bodies were negative, and an electrocardiogram indicated normal sinus rhythm (84 bpm).

Premedication with peroral antibiotics was carried out to prevent systemic infections that can be derived as a complication of DM. The patient’s initial postprandial blood glucose value just before surgery was 90 mg/dL. Preoperative cardiorespiratory parameters showed systolic/diastolic blood pressure of 162/93 mmHg, heart rate of 90 bpm, and oxygen saturation (SpO_2_) of 98%. Owing to the high blood pressure, the patient was treated to control intraoperative hypertension, with diligent attention to cardiovascular conditions; this was performed under the auspices of the first author, who is a certified dental anesthesiology specialist. An intravenous line with saline fluid was inserted for intravenous administration of nicardipine and/or diltiazem as antihypertensive agents to control blood pressure with noninvasive monitoring, including a lead II electrocardiogram. Local anesthesia with 3% prilocaine containing felypressin (0.03 IU/mL) as a vasoconstrictor for surgical procedures was applied to avoid unstable hemodynamics. Intravenous nicardipine (0.4 mg) and diltiazem (5 mg) were intermittently administered via a bolus injection to achieve a systolic blood pressure level lower than 150 mmHg with good control and stability of hemodynamics.

During surgery, the patient abruptly complained of discomfort such as malaise that seemed to be a symptom of hypoglycemia. At that time, neither conscious nor cardiorespiratory disturbance was confirmed, with blood pressure of 160/75 mmHg, heart rate of 75 bpm, and SpO_2_ of 96%. Blood glucose was promptly measured at 32 mg/dL and recognized as severe hypoglycemia. Oral glucose and an electrolyte-combined infusion of glucose were administered, and he immediately recovered, with blood glucose increasing to 65 mg/dL 15 min after glucose administration and to 127 mg/dL by the end of the surgical procedure.

**Fig. 1:**
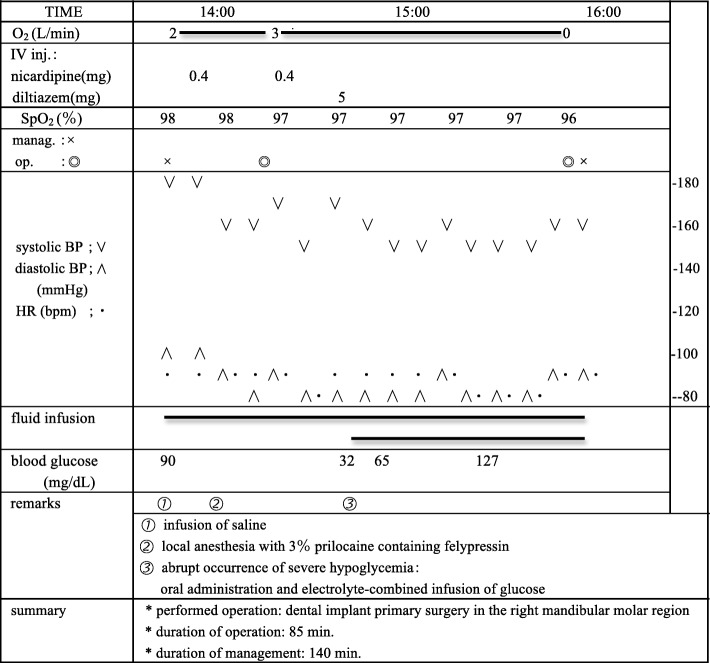
Intraoperative management record indicating the progress of glycemic control. During dental implant surgery, the patient abruptly complained of discomfort such as malaise that seemed to be a symptom of hypoglycemia. Blood glucose was promptly measured at 32 mg/dL and recognized as severe hypoglycemia. Oral glucose and an electrolyte-combined infusion of glucose were administered, and he immediately recovered, with blood glucose increasing to 65 mg/dL 15 min after glucose administration and to 127 mg/dL by the end of the surgical procedure

The present surgery, involving the placement of a screw-shaped endosseous implant fixture made of titanium in the lower jaw, was smoothly performed precisely as planned. There was no implant placement supplemented by various guided bone regeneration, and no other issues occurred. The surgical procedure resulted in less invasion, limited to the area of implant fixture placement within the right mandibular region of the two molars, compared to commonly performed dental implant surgery that spans the entire lower jaw and is likely to be relatively highly-invasive. The durations of surgery and systemic management were 85 min and 140 min, respectively (Fig. 1).

## Discussion

In patients with type 1 DM, pancreatic β-cells are destroyed, and endogenous insulin secretion capacity is depleted [2]. In comparison with type 2 DM, type 1 DM is characterized by poor glucose tolerance owing to insufficient secretion of endogenous insulin. This situation can promote the hypersecretion of catabolic hormones during invasive surgery or anesthesia and initiate a neuroendocrine stress response. The current case supports that even in the perioperative management for oral surgical outpatients, oral surgeons and dental anesthesiologists need to keep in mind the appropriate continuous administration of insulin with a glucose-electrolyte infusion and careful blood glucose monitoring [3].

From the viewpoint of prevention of diabetic complications, including ketoacidosis, development of systemic vascular lesions, and compromised infections or protracted wound healing, it is essential to conduct a systemic assessment based on diagnostic and prognostic criteria. Regarding preoperative blood glucose control for diabetic surgical patients, such assessments comprise negative urinary ketone bodies, HbA1c level lower than 7%, and a fasting blood glucose level lower than 130 mg/dL [4]. At blood glucose levels above 200 mg/dL, bacterial infectivity is promoted due to impairment in neutrophil phagocytosis [5], followed by postoperative wound healing failure. In addition, to avoid unexpected hypoglycemia and postoperative infections in oral surgical patients, it is also recommended that blood glucose level should be maintained at approximately 150 mg/dL [6]. If HbA1c is lower than 7% but exceeds 6.2% (upper limit of normal range), premedication with antibiotics is considered necessary owing to an increased risk of focal infections [7].

As shown in the present case, medical control of type 1 DM is difficult owing to highly variable diurnal or day-to-day variation in blood glucose levels. Furthermore, even immediately after typical food intake, a large variation of blood glucose may be irregularly triggered by psychosomatic stress related to dental surgical procedures. On the other hand, to differentiate hypoglycemic symptoms from other clinical signs, it is important to be well acquainted with some principal clinical symptoms characteristic of hypoglycemia as follows. If blood glucose level decreases below 70 mg/dL, sympathetic stimulation-related symptoms such as abnormal hunger, anxiety, palpitation, cold sweat, and/or tremor can be developed in conjunction with catecholamine hypersecretion. In a hypoglycemic situation, when blood glucose level is below 50 mg/dL, nausea, malaise, drowsiness, headache, delirium, visual abnormality, and/or bradycardia may appear owing to suppression of central nervous function. Hypoglycemia below 30 mg/dL results in convulsions and/or coma due to cerebral dysfunction [8, 9].

During the present perioperative management, in consideration of diabetes, an electrolyte solution containing rapid acting insulin was prepared according to the sliding scale of our hospital for blood glucose values above 200 mg/dL. In the present patient, who had experienced repeated hypoglycemic attacks, signs of hypoglycemia, without any sympathetic symptoms, may be specifically recognized only when blood glucose level decreases below 30 mg/dL. Accordingly, prompt subcutaneous injection of glucagon [10] may be also required, based on the appearance of conscious disturbance, which is a hypoglycemic sign. Thus, to ensure glycemic control, we recognized that we should have preoperatively prepared the appropriate administration of insulin with an electrolyte-combined infusion of glucose.

The application of felypressin as a vasoconstrictor for local anesthesia in the present patient was judged to be appropriate to avoid a hypertensive emergency and hyperglycemia due to extrinsic adrenaline. Moreover, considering that systemic blood flow disorders in patients with diabetes and hypertension may extend to the weakened microvessels of periodontal tissues [11], we were concerned about the latent risk of gingival ulceration resulting from strong vasoconstriction.

Patients undergoing dental implant surgery are likely to have various systemic underlying diseases such as DM or hypertension. Consequently, the clinical insights and skills regarding medication are essential to practice safe perioperative management of blood glucose or hemodynamics. Furthermore, patients with type 1 DM may not manifest typical hypoglycemia symptoms, despite a severely low blood glucose level. In this regard, we reaffirmed the importance of a more careful time-series evaluation of blood glucose in perioperative glycemic management for the present patient undergoing dental implant surgery.

In the light of the above viewpoints, it seems more difficult to manage a patient such as the present case owing to limited use of cardiorespiratory monitoring devices and limited care or medication for an emergency involving severe hypoglycemic attack or unstable hemodynamics under normal private practice conditions. Therefore, we suggest that it might be advisable to operate on such a patient in an institution that can provide satisfactory cardiorespiratory monitoring equipment and medication, taking into consideration the high-risk complications including severe hypoglycemia and hypertension.

In conclusion, although the surgical procedure for this patient with type 1 DM was less invasive and limited to the area of implant manipulation, within the mandibular region of the two molars, compared to implant surgery that spans the entire lower jaw, the present case suggests the necessity of examining possible signs of hypoglycemia-precipitated acute symptoms in patients with DM. This is particularly true in patients with type 1 DM who are undergoing relatively highly-invasive oral surgical manipulation such as commonly performed dental implant surgery spanning the entire jaw. It is also important to accomplish appropriate emergency care, differentiating hypoglycemic symptoms from other clinical signs. Therefore, dental anesthesiologists as well as oral surgeons are specifically required to provide principal cardiorespiratory monitoring and carry out careful systemic management particularly for a medically compromised patient such as the one we have presented with type 1 DM who may experience complications including severe hypoglycemia and unstable hemodynamics.

## Abbreviations

DM: Diabetes mellitus
HbA1c: Glycated hemoglobin
SpO_2_: Oxygen saturation
